# Correlates of polyneuropathy in Parkinson’s disease

**DOI:** 10.1002/acn3.51182

**Published:** 2020-09-17

**Authors:** Eva Kühn, Paulina Averdunk, Sophie Huckemann, Katharina Müller, Anne‐Sophie Biesalski, Florian Hof zum Berge, Jeremias Motte, Anna Lena Fisse, Christiane Schneider‐Gold, Ralf Gold, Kalliopi Pitarokoili, Lars Tönges

**Affiliations:** ^1^ Department of Neurology St. Josef‐Hospital Ruhr‐University Bochum Germany; ^2^ Neurodegeneration Research Centre for Protein Diagnostics (ProDi) Ruhr‐University Bochum Germany

## Abstract

**Objective:**

Previous studies in Parkinson’s disease (PD) patients have demonstrated a high prevalence of polyneuropathy (PNP) and pronounced alpha‐Synuclein pathology in dermal nerve fibers already at early disease stages. The aim of this study was to analyze associations between the prevalence and severity of PNP with nonmotor and motor symptoms in PD patients.

**Methods:**

Fifty PD patients were characterized comprehensively for the presence of clinical symptoms (nonmotor and motor), electrophysiologic alterations and – for the first time – using high‐resolution ultrasound of peripheral nerves.

**Results:**

Sixty‐two percent of PD patients showed electrophysiological pathology of PNP. The prevalence of patient‐reported PNP symptoms was 86% and was particularly present in patients with longer disease duration, compromised scores of nonmotor and motor symptoms as well as with a negative evaluation of quality of life. Seventy‐five percent of patients showed morphologic alterations similar to axonal PNP in high‐resolution ultrasound compared to healthy controls.

**Interpretation:**

The study demonstrates the high burden of peripheral nervous system disease in Parkinson's disease. It advocates further studies to delineate the underlying pathophysiological mechanisms in order to optimize treatment approaches for PD, including the associated PNP.

## Introduction

Parkinson’s disease (PD) is the most common chronic neurodegenerative movement disorder and presents with both motor and nonmotor symptoms.^1^ Nonmotor features include sleep alterations, dementia, depression, constipation, and pain that may severely compromise quality of life and may conversely influence the burden of motor symptoms including gait.^2^ Polyneuropathy (PNP) is a frequent but often underestimated complication of PD. There are very much varying estimations about the overall prevalence of PNP in PD which is between 19% and 55% while only 8% to 9% of age‐related peers are affected.^3^, ^4^ The characteristics of PD‐associated PNP are only insufficiently studied. This is in part due to heterogeneous study designs with differing thoroughness of clinical and paraclinical evaluation but also due to lack of sufficient control for confounding variables such as age, diabetes, or alcohol consumption.^5^ The influence of levodopa utilization and pathological changes in cobalamin, folate, and homocysteine levels have been discussed controversially,^3^, ^6^, ^7^ but additional factors are also suspected as several studies have demonstrated dermal nerve involvement in PD including the presence of alpha‐Synuclein (aSyn) pathology in clinically manifest but also in prodromal states.^8^ In this observational analysis we systematically study and compare clinical and electrodiagnostic features of nerve involvement in a monocentric cohort of PD and – for the first time – apply noninvasive high‐resolution nerve ultrasound (HRUS) diagnostics to evaluate morphologic substrates of PNP in PD.

## Subjects and Methods

**Figure 1:**
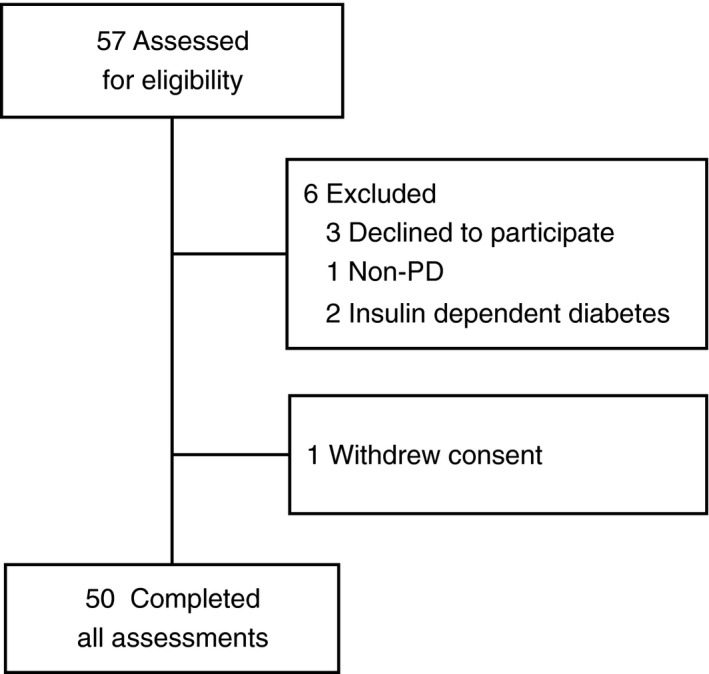
Study flow diagram.

Between October 2018 and April 2019, we assessed 57 PD patients from the Department of Neurology in St. Josef Hospital of Ruhr‐University Bochum for eligibility who fulfilled both United Kingdom Parkinson's Society Brain Bank criteria and Movement Disorders Society's Criteria for Parkinson's disease.^9^, ^10^ Three patients declined to participate in the study, three patients were excluded as they did not fulfill our inclusion criteria and one patient withdrew consent during the study (Fig. 1).

Main exclusion criteria were known diagnosis of diabetes, alcohol abuse, and other known causes of neuropathy. In addition, patients with severe dementia, patients with insufficient language skills or illiterate persons and patients with acute mental disorders were excluded from the study. All patients provided written informed consent before enrollment.

Ethical approval was given by the Ethics Committee of the Medical Faculty of the Ruhr University Bochum (register‐number 18–6360, date of approval 12.09.2018). The study was performed in accordance with the ethical standards of the Declaration of Helsinki and was registered in the German Clinical Trials Register (ID: DRKS00020752). Data were saved in a pseudonymized way and were processed according to local legal conditions.

### Clinical assessments

Clinical examination comprised patient interview, neurological examination, modified Neuropathy Disability Score (NDS),^11^, ^12^ and documentation of the following scores: MDS‐Unified Parkinson's Disease Rating Scale (MDS‐UPDRS) Part I‐IV,^13^ Assessment of Autonomic Dysfunction in Parkinson Disease (SCOPA‐AUT),^14^ Nonmotor Symptom Questionnaire (NMSQuest),^15^ Parkinson's Disease Questionnaire (PDQ‐39),^16^ and modified Neuropathy Symptom Score (NSS) (Table 1).^11^

**Table 1:** Clinical examination of the study population.

Routine examination	Clinical PD scores	Clinical PNP scores
Patient Interview Neurological examination Test of: achilles tendon, patellar tendon, biceps tendon, triceps tendon and brachioradial reflex Vibration detection threshold Functional test of cranial nerves	MDS‐Unified Parkinson's Disease Rating Scale (MDS‐UPDRS) Part I‐IV Assessment of Autonomic Dysfunction in Parkinson Disease (SCOPA‐AUT) Nonmotor Symptom Questionnaire (NMSQuest) Parkinson's Disease Questionnaire (PDQ‐39)	Modified Neuropathy Symptom Score (NSS) Modified Neuropathy Disability Score (NDS)

### Nerve conduction studies, HRUS, and diagnosis criteria of PNP

All patients underwent electrophysiological examination performed by a board‐certified neurologist with the use of a Medtronic four channel electroneurography device (Medtronic, Meerbusch, Germany). Motor studies of tibial and median nerve as well as sensory studies of sural and median nerve were done bilaterally maintaining skin temperature at 36°C and were referenced to normal values.^17^ Nerve HRUS examination was performed with an Affinity^®^70G ultrasound system (Philips, Hamburg, Germany) with an 18‐MHz linear array transducer as described previously. It was performed bilaterally at entrapment and nonentrapment sites. The entrapment sites included the median nerve (Carpal tunnel), the ulnar nerve (Loge de Guyon and Sulcus ulnaris), and fibular nerve (Caput fibularis). The nonentrapment sites contained the median nerve (upper arm), the ulnar nerve (upper arm), and the fibular nerve (fossa tibialis). In order to avert anisotropy, the transducer was kept perpendicular to the nerves and no additional force was applied while the extremities were kept in neutral position to avoid nerve deformation. The measurement of CSA was performed at the inner border of the thin hyperechoic epineural rim by a continuous tracing technique.

The diagnosis criteria for PNP were determined by nerve conduction studies. The lower value of bilateral conduction was regarded to detect early PNP. In order to include and analyze neuropathy groups with different severity in NCS we defined three PNP subgroups based on normal values^17^ (Table S1).

The criteria for mild, sensory PNP were an amplitude of the sural sensory nerve action potential (sNAP) <5 mV for patients <50 years or <3.6 mV for patients older than 50 years, and an amplitude of the tibial compound motor nerve action potential (cMAP) >5 mV and an amplitude of the median cMAP >5 mV. The criteria for moderate, sensorimotor PNP were an amplitude of the sural sNAP <5 mV for patients <50 years and <3.6 mV for patients older than 50 years and an amplitude of the tibial cMAP <5 mV and an amplitude of the median cMAP >5 mV.

The criteria for severe, sensorimotor PNP were an amplitude of the sural sNAP <5 mV for patients <50 years and <3.6 mV for patients older than 50 years and an amplitude of the tibial and median cMAP <5 mV.

### Laboratory assessment

All patients underwent a laboratory examination for blood cell count, HbA1c, liver enzymes, urea, electrolytes, creatinine, thyroid stimulating hormone, vitamin B12, B1, B6, methylmalonic acid, homocysteine, holotranscobalamin levels, and serum protein electrophoresis/immunfixation to rule out other causes for a peripheral neuropathy.

### Statistical analysis

Clinical, demographic, and electrophysiological data are presented as means with standard deviations. Differences between groups of PNP were calculated by Student’s *t*‐test or ANOVA (normally distributed variables) or Mann–Whitney *U* test or Kruskal–Wallis test (not normally distributed variables). Correlations between electrophysiological measures and clinical parameters were calculated based on Pearson’s *r* and multiple linear regression analyses. Global significance was set at α < 0.05 and a Bonferroni–Holm correction was performed for each hypothesis in order to reduce the probability of error of the first kind through multiple testing.^18^ Correlations, which did not reach the significance level after Bonferroni–Holm correction are specifically mentioned in the results section. Statistical analyses were performed with SPSS version 25.0 (IBM Deutschland GmBH, Ehningen, Germany).

### Standard protocol approvals and registration

The Ruhr‐University Institutional Review Board approved this study (Reg. Nr. 18‐6360). It is listed in the German Clinical Trials Register (DRKS‐ID DRKS00020752).

## Results

### Clinical and demographic data of PNP subgroups

Fifty PD patients (24 female and 26 male patients, mean age 67.8 ± 10.4 years) were included into the analysis with a mean disease duration of 6.5 ± 5.1 years, mean levodopa dosage (LED) of 590 ± 391, and mean MDS‐UPDRS III of 31.2 ± 16.6. Of the 50 patients, 31 patients fulfilled the electrodiagnostic criteria for PNP. Fourteen patients had a mild, sensory PNP, 11 patients had a moderate, sensorimotor PNP. Six patients had a severe, sensorimotor PNP.

Importantly, concerning PNP subgroups, there were no statistically significant differences between sexes, age of onset or for LED observed (Table 2).

**Table 2:** Demographics, clinical and instrument‐based analysis of PD patients and classification of subgroups with polyneuropathy and average values of high‐resolution ultrasound examination in PD patients with/without PNP and non‐PD patients (healthy controls) at entrapment sites and non‐entrapment sites.

	Total PD patients (*n* = 50)	PD patients without PNP (*n* = 19)	PD patients with PNP (*n* = 31)	PD patients with mild/sensory PNP (*n* = 14)	PD patients with moderate/sensorimotor PNP (*n* = 11)	PD patients with severe/sensorimotor PNP (*n* = 6)	Healthy controls (Non‐PD) (*n* = 75)
Mean age at evaluation ± SD (years)	67.8 ± 10.4	62 ± 8.5	71.3 ± 10	68.9 ± 9.6	69.6 ± 11	80.2 ± 2.9	Female: 47.5 ± 14.3 Male: 40.8 ± 16.2
Female	24	11	13	9	2	2	
Disease duration ± SD (years)	6.5 ± 5.1	5 ± 3.7	7.5 ± 5.7	7.1 ± 5	5.7 ± 4.8	11.5 ± 7.6	
Mean age at PD diagnosis ± SD (years)	61.2 ± 9.7	57 ± 8.4	63.9 ± 9.6	61.9 ± 8.8	63.8 ± 11.2	68.7 ± 7.5	
Mean Levodopa equivalence dose ± SD	590 ± 391	546 ± 428	617 ± 372	564 ± 409	609 ± 284	754 ± 450	
Mean Hoehn and Yahr Score ± SD	2.8 ± 0.8	2.5 ± 0.7	3 ± 0.9	2.9 ± 0.9	2.9 ± 1	3.3 ± 0.8	
Mean MDS‐UPDRS III Score ± SD	31.2 ± 16.6	21.6 ± 13.9	37.5 ± 15.8	33.8 ± 14	36.6 ± 15.4	47.7 ± 17.6	
Mean NMSQuest Score ± SD	10.6 ± 5.9	8.6 ± 5.5	11.8 ± 5.8	9.7 ± 4.8	12.5 ± 5.7	15.7 ± 6.8	
Mean SCOPA‐AUT Score ± SD	14.8 ± 9.1	12 ± 9.2	16.5 ± 8.8	13.9 ± 5.6	18.6 ± 10.4	18.4 ± 11.3	
Mean PDQ‐39 Score ± SD	46.6 ± 30.6	39.8 ± 28.3	50.7 ± 31.9	45.4 ± 26.2	50 ± 38.9	64.5 ± 31.9	
Mean NSS Score ± SD	5 ± 2.9	4 ± 3.3	5.7 ± 2.6	5 ± 2.4	6 ± 3	6.7 ± 1.7	

Mean phenotype distribution	TD = 18 PIGD = 28 interm. = 4	TD = 9 PIGD = 9 interm. = 1	TD = 9 PIGD = 19 interm. = 3	TD = 3 PIGD = 10 interm. = 1	TD = 5 PIGD = 5 interm. = 1	TD = 1 PIGD = 4 interm. = 1	
NCS: mean amplitude (mV)							
N. suralis	3.1 ± 3.7	6.8 ± 3.4	0.8 ± 1	1.4 ± 1.1	0.4 ± 0.9	0.3 ± 0.3	17.2
N. tibialis	7 ± 4.2	10.1 ± 4	5.1 ± 3	7.7 ± 2	3.4 ± 1.6	2.1 ± 1.8	19.1
N. medianus (motor)	5.4 ± 1.7	5.9 ± 1.2	5.2 ± 1.9	6 ± 1.1	6.1 ± 0.8	2.2 ± 1.4	13.2
N. medianus (sensory)	9.6 ± 7.2	12.7 ± 8	7.9 ± 6.4	10.3 ± 7.9	7.5 ± 4.6	4 ± 3.1	13.7
HRUS: mean CSA of entrapment sites (mm^2^)							
N. medianus (Carpal tunnel)	10.3 ± 2.3	9.8 ± 2	10.7 ± 2.5	10.3 ± 7.9	10.9 ± 2.1	10.2 ± 1.4	8.4 ± 2
N. ulnaris (Loge de Guyon)	6.8 ± 1.6	6.7 ± 1.3	6.8 ± 1.8	6.8 ± 2	7.2 ± 1.7	6.1 ± 1.6	5.2 ± 1
N. ulnaris (Sulcus ulnaris)	7.6 ± 1.8	7.4 ± 1.3	7.7 ± 2.1	7.8 ± 2.7	7.6 ± 0.6	8.0 ± 2.4	5.3 ± 1.4
N. fibularis (Caput fibularis)	13.9 ± 5.7	12.4 ± 3.4	15 ± 6.7	15.9 ± 5.9	16.2 ± 8.7	10.7 ± 3.2	7.1 ± 2.3
HRUS: mean CSA of non‐entrapment sites (mm^2^)							
N. medianus (upper arm)	9.4 ± 2.6	8.8 ± 2.1	9.8 ± 2.9	10.1 ± 3.6	9.8 ± 2.1	8.8 ± 2.5	8.4 ± 2.1
N. ulnaris (upper arm)	6.2 ± 2	5.9 ± 1.2	6.3 ± 2.4	6.8 ± 3.1	5.8 ± 1.4	6 ± 2.2	6.5 ± 1.8
N. fibularis (fossa tibialis)	9.6 ± 4.2	8.2 ± 2.1	10.7 ± 5	11 ± 4.8	11.9 ± 5.9	7.1 ± 1.8	8.4 ± 2.7

Criteria for mild/sensory PNP: amplitude of the sural nerve <5 mV for patients <50 years and <3.6 mV for patients older than 50 years, amplitudes of the tibial and median nerve >5 mV. Criteria for moderate/sensorimotor PNP: amplitude of the sural nerve <5 mV for patients <50 years and <3.6 mV for patients older than 50 years, amplitude of the tibial nerve <5 mV and amplitude of the median nerve >5 mV. Criteria for severe/sensorimotor PNP: amplitude of the sural nerve <5 mV for patients <50 years and <3.6 mV for patients older than 50 years, amplitudes of the tibial and median nerve <5 mV. CSA, cross‐sectional area; interm., intermediate type; MDS‐UPDRS, MDS‐Unified Parkinson's Disease Rating Scale; NSS, Neuropathy Symptom Score; NMSQuest, Nonmotor Symptom Questionnaire; PD, Parkinson's Disease; PDQ‐39, Parkinson's Disease Questionnaire; PIGD, postural instability and gait disorder; PNP, polyneuropathy; SCOPA‐AUT, Assessment of Autonomic Dysfunction in Parkinson Disease; TD, tremor dominant.

### Patient‐reported neuropathy symptoms correlate with motor‐ and nonmotor symptoms in PD

The subjective perception of functional deficits plays an important role for both sensory and motor symptoms in all disease stages of PD.^19^ We have quantified patient‐reported PNP symptoms with the NSS and correlated it to motor and nonmotor symptom scores.

**Figure 2:**
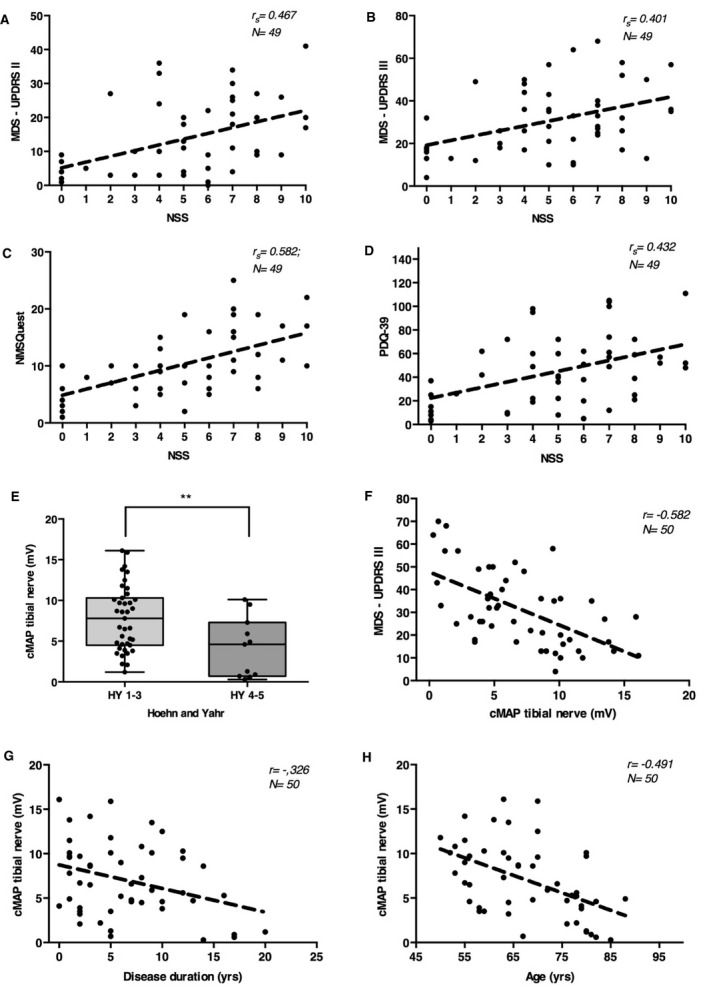
(A) MDS‐UPDRS III in relation to NSS (*r*_s_ = 0.401; *P* = 0.002; *n* = 49). (B) MDS‐UPDRS II in relation to NSS (*r*_s_ = 0.467; *P* < 0.001; *n* = 49). (C) NMSQuest in relation to NSS (*r*_s_ = 0.582; *P* < 0.001; *n* = 49). (D) PDQ‐39 in relation to NSS. (*r*_s_ = 0.432; *P* = 0.001; *n* = 49). (E) Amplitude of the tibial nerve in Hoehn and Yahr Groups (*n* = 50). (F) MDS‐UPDRS III in relation to the amplitude of the tibial nerve (*r* = −0.582, *P* < 0.001; *n* = 50). (G) Amplitude of the tibial nerve in relation to disease duration (*r* = −0.326; *P* = 0.01; *n* = 50). (H) Amplitude of the tibial nerve in relation to age at the time of examination (*r* = −0.49; *P* < 0.001; *n* = 50). MDS‐UPDRS, MDS‐Unified Parkinson's Disease Rating Scale; PDQ‐39, Parkinson's Disease Questionnaire.

The mean MDS‐UPDRS III score was higher in patients with more severe symptoms of PNP and scoring of at least 6 points on the NSS (NSS 0‐5 with mean MDS‐UPDRS III of 27, NSS 6‐10 with mean MDS‐UPDRS III of 34.7; *P* = 0.045; not significant after Bonferroni–Holm Correction [α = 0.0083]). NSS significantly correlated with the MDS‐UPDRS III value (*r*_s_ = 0.401; *P* = 0.004; *n* = 49) and also with the self‐reported MDS‐UPDRS II (*r*_s_ = 0.467; *P* = 0.001; *n* = 49), the latter being independent of patient age (Fig. 2A and B) what shows us a fair relationship.^20^ Forty‐two patients reported at least one neuropathic symptom in the NSS. No patient reported neuropathic symptoms without asking directly.

We see a correlation between NSS and nonmotor symptoms as evaluated with the NMSQuest (*r*_s_ = 0.600; *P* < 0.001; *n* = 49) as well as the correlation to life quality as evaluated with the PDQ‐39 (*r*_s_ = 0.432; *P* = 0.001; *n* = 49), both being independent of age (Fig. 2C and D).

### Peripheral nerve motor amplitudes correlate with disease severity and motor symptoms

The electrophysiological examination is the gold standard in instrument‐based detection and longitudinal monitoring of PNP. In order to correlate electrophysiology‐based analysis of sensory and motor peripheral nerve function in PD we selected the distal tibial compound muscle action potential (cMAP) and the sural sensory nerve action potential (sNAP). The sural sNAP did not correlate with most of clinical assessments of PD as the sural sNAP amplitudes were very low in the majority of patients (Fig. S1).

There was a significant difference of the sural sNAP between patients in lower Hoehn and Yahr stages (Hoehn and Yahr 1‐3) and more advanced stages (Hoehn and Yahr 4‐5) (*P* = 0.001; *n* = 50) (Fig. S1A). The inverse correlation of sural sNAP with MDS‐UPDRS III was not independent of age (β [age] = 0.578, *P* < 0.001; β [sural sNAP] = −0.202, *P* = 0.100) (Fig. S1B). There was no correlation of sural sNAP and disease duration (*r*_s_ = −0.243; *P* = 0.088; *n* = 50) (Fig. S1C).

The mean tibial nerve cMAP value was significantly lower in advanced Hoehn and Yahr stages of at least 4 than in less advanced stages (mean = 4.19 mV vs. 7.80 mV; *P* = 0.005; *n* = 50) (Fig. 2E). If tibial nerve cMAP and MDS‐UPDRS III values were analyzed, this showed a strong inverse correlation with lower tibial cMAP indicating higher motor symptom severity being independent of age or LED (*r* = −0.582, *P* < 0.001; *n* = 50) (Fig. 2F). Interestingly, the tibial nerve cMAP also correlated with overall disease duration, the cMAP being lower with a longer disease course (*r* = −0.326; *P* = 0.021; *n* = 50), and with age in general (*r* = −0.491; *P* < 0.001; *n* = 50) (Fig. 2G and H).

### Morphologic variability of nerve pathology in high‐resolution ultrasound of peripheral nerves in PD

**Figure 3:**
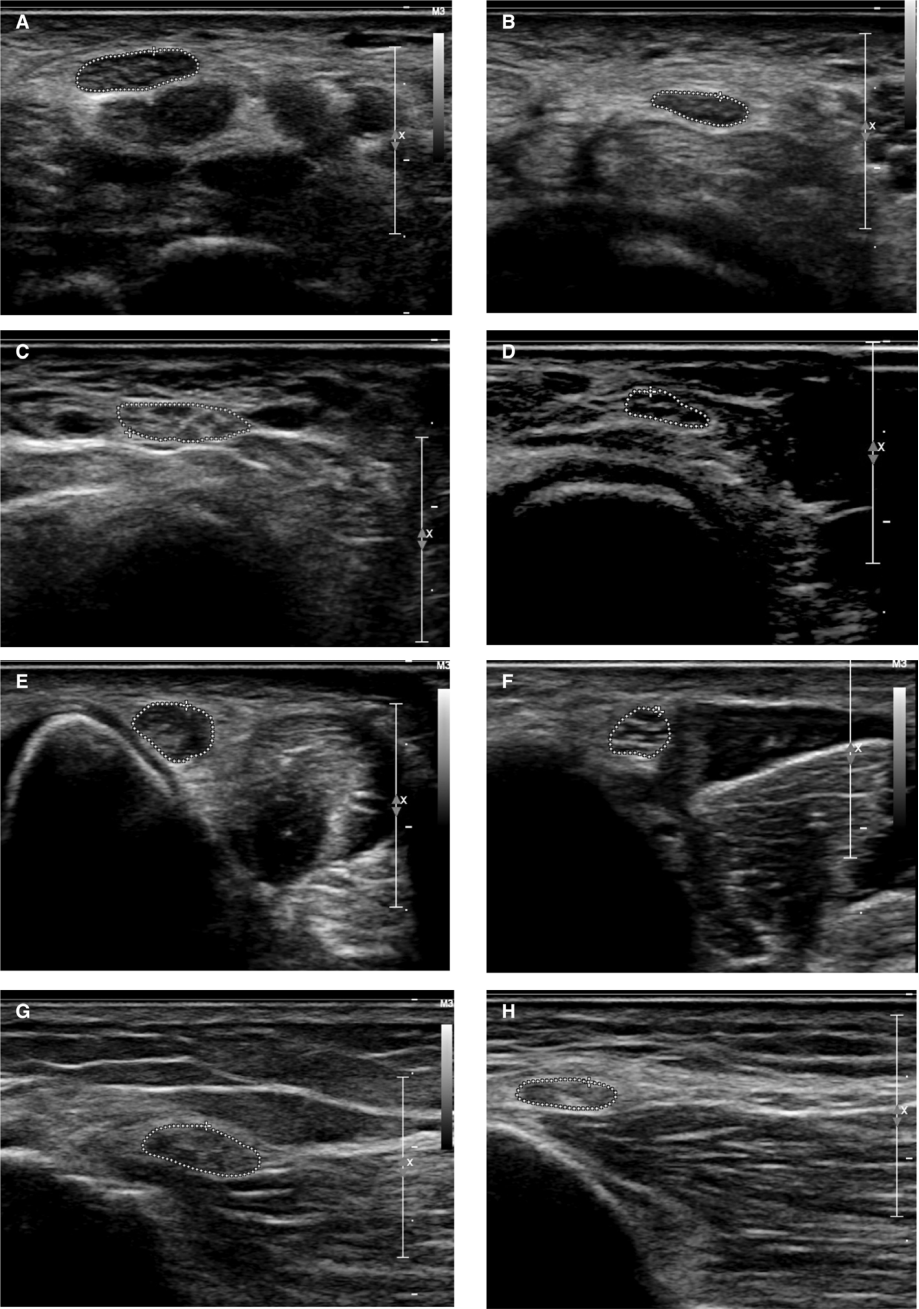
(A–H) High‐resolution ultrasound of peripheral nerves at entrapment sites: PD patients (A, C, E, and G; for all *n* = 45) versus controls (B, D, F, and H; for all *n* = 75): A and B: median nerve: carpal tunnel. (C and D) Ulnar nerve: Loge de Guyon. (E and F) Ulnar nerve: sulcus. (G and H) Fibular nerve: head. PD, Parkinson’s disease.

**Figure 4:**
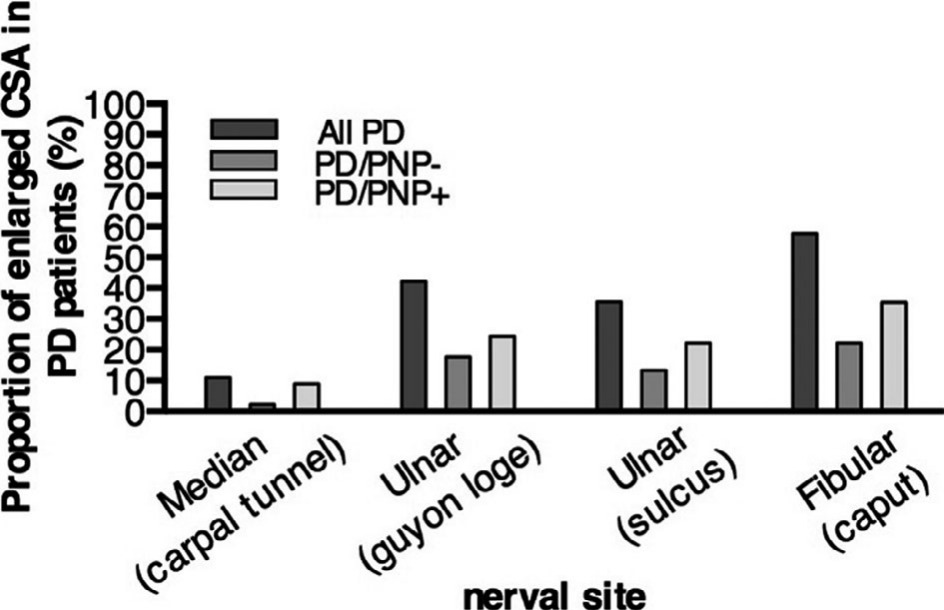
Proportion of PD patients with an increased CSA in 45 patients with (PD/PNP+) or without (PD/PNP−) diagnosis of polyneuropathy in nerve conduction study at entrapment points. PD, Parkinson’s disease; CSA, cross‐sectional area; PNP, polyneuropathy.

HRUS is a recently developed imaging method which very precisely detects morphological alterations of peripheral nerves.^21^ We applied HRUS technology for the in vivo examination in peripheral nerves in PD. First, we examined entrapment sites where peripheral nerve pathology can be pronounced because of constantly higher mechanical burden. Four of 45 PD patients showed enlarged cross‐sectional areas (CSAs) of the median nerve at the carpal tunnel larger than 12.6 mm^2^ in comparison to 75 healthy control values.^22^ Concerning the ulnar entrapment site at the Loge de Guyon, 19 of 45 patients showed enlarged CSA (>7.2 mm^2^) and in the ulnar sulcus entrapment site, 16 of 45 patients had enlarged values (>8.1 mm^2^). The entrapment site CSA of leg fibular nerve demonstrated 26 of 44 patients with enlarged nerves (>11.7 mm^2^) (Figs. 3, 4).

At nonentrapment sites of peripheral nerves, only few patients showed morphological alterations. Six of 45 PD patients showed enlarged CSA of the median nerve at the upper arm (>12.54 mm^2^), 2 of 45 PD patients showed enlarged CSA of the ulnar nerve at the upper arm (>10.17 mm^2^) and 6 of 44 PD patients showed enlarged CSA of the fibular nerve in the popliteal fossa (>13.79 mm^2^). High‐resolution ultrasound alterations and nerve conduction studies did not correlate to a significant extent. Only the CSA of the median nerve at the upper arm showed a weak but significant correlation with the tibial nerve cMAP (*r*_s_ = 0.317; *P* = 0.049; *n* = 39) (Table S2). As we proved that PNP presents in PD with a high prevalence, we show for the first time that the type of PNP is a typical axonal PNP as evaluated by ultrasound and does not present with specific focal enlargement distribution as shown for demyelinating polyneuropathies.

### Laboratory assessment of PNP in PD

In addition, we performed a broad analysis of serum parameters that could be associated with PNP. There were no age independent effects for other etiologies of PNP such as deficits of folate, cobalamin, methylmalonic acid, and homocysteine. Of interest, when age was included as a confounder LED did not correlate significantly with tibial nerve cMAP (β = −0.219, *P* = 0.074) or with HRUS pathology (data not shown).

## Discussion

Our clinical evaluation of peripheral nervous system (PNS) involvement in PD revealed a high presence of subjective complaints with 86% of patients reporting at least one neuropathic symptom. Nerve conduction studies – which generally serve as gold standard for the diagnosis of PNP – proved to be significantly abnormal in 62% of patients compared to up to 55% in previous reports^3^, ^4^ which indicates the high PNS lesion burden in PD.

In a previous study, the prevalence of PNP was shown to be 30.9% (Crespo‐Burillo et al.). Here, a positive correlation between Hoehn and Yahr stage with the age of onset of PD and presence of PNP was described. Therefore, more advanced disease seems to have an influence on the severity of PD‐associated PNP symptoms. While the age at PD onset was quite comparable between the two cohorts (61.4 ± 10.3 in Crespo‐Burillo; 61.2 ± 9.7 in our cohort), in our study population, the Hoehn and Yahr stage showed a higher mean value of 2.8 ± 0.8 (Crespo‐Burillo 2.1 ± 0.7) which could explain the higher prevalence of PNP.^23^ The study by Toth et al. found a prevalence rate of 58% in 58 PD patientens by applying the AAN clinical research criteria for distal symmetric PNP.^3^ These included abnormal nerve conduction studies of at least the sural and peronal nerve, as well as neuropathic symptoms (Toronto clinical scoring system) and clinical signs (Neuropathy Impairment Score). As our PNP criteria also included at least one pathological finding in bilateral nerve conduction studies of the sural nerve, this would explain similar prevalence rates. In addition, UPDRS III scores were slightly more elevated higher in the Toth et al. cohort (42.1 ± 27.9) compared to ours (31.2 ± 16.6) and disease duration of PD was longer (8.8 ± 9.5 vs. 6.5 ± 5.1).^3^ In an Italien study, the PNP prevalence rate was only 11.8% in 330 PD patients.^7^ However, that study population demonstrated much less motor impairment (UPDRS III = 19.19) and the diagnostic criteria for PNP differed with nerve conduction studies being performed unilaterlly only.^7^

Patients with longer disease duration, compromised scores of nonmotor (NMSQuest) and motor symptoms (MDS‐UPDRS III) as well as negative evaluation of quality of life (PDQ‐39) reported significantly more subjective complaints of PNP. Therefore, our findings are in alignment with a previous study that found associations between the severity of PNP symptoms and motor symptoms of PD.^3^ Abnormal nerve conduction studies, in particular reduction of tibial nerve cMAP, reflected the severity of clinical burden related to motor symptoms revealed by MDS‐UPDRS III analysis. PD motor burden correlated more with age and with cMAP amplitude reduction of the tibial nerve than with disease duration per se suggesting that this electrophysiological parameter could be a marker for severity of motor symptoms. In analogy to the concept of subjective cognitive decline in dementia^24^ the perception of minor sensory symptoms in PD patients could indicate an involvement of the PNS which is not yet detectable by nerve conduction studies. A longitudinal study with a larger cohort, PD and non‐PD patients is needed.

A high proportion of PD patients showed enlarged CSAs at neural entrapment sites (Carpal tunnel, Loge de Guyon, sulcus ulnaris, fibular head) as examined with HRUS in comparison to controls. Importantly, the distribution of HRUS abnormalities differs from abnormalities in inflammatory demyelinating diseases like chronic inflammatory demyelinating polyneuropathy (CIDP). In CIDP, previous studies revealed an increase of CSA of the ulnar nerve at the Loge de Guyon, upper arm, of the radial nerve in upper arm, of the sural nerve, and of the distal tibial nerve. In the present study, however, we detected increased CSA mostly at typical entrapment sites such as the fibular head and the ulnar sulcus without obvious local clinical symptoms nor enhanced electrophysiological alterations.^25^ This could imply that nerves in PD patients are more vulnerable and at higher risk to be lesioned due to mechanical irritation at entrapment sites. Enlargement of peripheral nerve CSAs in PD patient was found in both PNP positive and PNP negative cases. However, the prevalence was much higher in PNP positive patients. CSA increase in entrapment sites was not associated with relevant clinical entrapment symptoms. In our view, this could represent subclinical morphological alterations in entrapments sites similar to the findings in patients with other types of axonal neuropathy such as in diabetes or restless‐legs syndrome.^26^, ^27^ To support this hypothesis of increased nerve vulnerability in PD, more detailed histopathological examinations are required.

Several studies have demonstrated aSyn pathology in, for example pharyngeal but also in dermal nerves in PD patients in clinically manifest but also in prodromal states such as RBD.^8^ Here, immunoreactivity of total aSyn and phosphorylated aSyn is seen as a putative in vivo biomarker of disease and was shown to very precisely discriminate alpha‐Synucleinopathies such as PD and MSA from other parkinsonian syndromes with high sensitivity and specificity.^28^ In a very recent study, aSyn oligomers were also quantified in synaptic terminals of peripheral nerve autonomic fibers and were shown to be significantly increased in PD patients compared to healthy controls with a specificity of 82%.^29^ As progression of PD is supposed to involve spreading of pathology from the periphery to the CNS,^30^ the peripheral nerve involvement itself may also progress and aSyn pathology could increasingly compromise peripheral nerve function and lead to functional and morphologic alterations.^31^

Limitations of the presented study are the patient number and the monocentric, observational study design. A longitudinal study design can evaluate a progression of PNP in relation to the progression of PD symptoms. Also, the causal relationship of PNP in PD still remains unclear, which could be elucidated by immunohistologic or label‐free imaging analysis of the skin or peripheral nerves. As the examination technique of HRUS is available in only a few centers and has not yet been applied to PD patients, our analysis study was planned as a first pilot study to evaluate the presence of morphologic alterations of peripheral nerves in PD with ultrasound technology and would definitively benefit from a multicenter analysis, which is planned as a next step.

In summary, we here demonstrate that PNP has a high prevalence in PD patients and is associated with nonmotor and motor symptoms of PD as well as with disease severity. A prospective study design is needed to determine PNP progression in PD patients and the role of electrophysiological parameters to reflect PD progression. HRUS is capable to detect morphologic correlates of PNS involvement in PD. Its diagnostic value for the evaluation of PD‐associated PNP has still to be shown.

## Author Contributions

Eva Kühn and Paulina Averdunk were involved in acquisition of data, analysis and interpretation, writing of the manuscript, and critical revision of the manuscript for important intellectual content. Sophie Huckemann and Katharina Müller were involved in acquisition of data, analysis and interpretation, and critical revision of the manuscript for important intellectual content. Dr. Anne‐Sophie Biesalski, Florian Hof zum Berge, Dr. Motte, and Dr. Fisse were involved in acquisition of data, critical revision of the manuscript for important intellectual content. Dr. Schneider‐Gold was involved in critical revision of the manuscript for important intellectual content. Dr. Gold reports no disclosures and was involved in critical revision of the manuscript for important intellectual content. Dr. Pitarokoili and Dr. Tönges were involved in study concept and design, acquisition of data, analysis and interpretation, writing of the manuscript, and critical revision of the manuscript for important intellectual content.

## Conflict of Interest

Paulina Averdunk, Eva Kühn, Sophie Huckemann, Katharina Müller, Anne‐Sophie Biesalski, Florian Hof zum Berge, Dr. Motte, Dr. Schneider‐Gold, Dr. Gold, Dr. Pitarokoili, and Dr. Tönges report no disclosures. Dr. Fisse: owns shares of Fresenius SE & Co., Gilead Sciences, Medtronic PLC and Novartis AG.

## Supporting information


**Figure S1.** (A) Amplitude of the sural nerve in Hoehn and Yahr Groups (*n* = 50). (B) MDS‐UPDRS III in relation to the amplitude of the sural nerve (*r*_s_ = −0.519, *P* < 0.001; *n* = 50). (C) Amplitude of the sural nerve in relation to disease duration (*r*_s_ = −0.243; *P* = 0.088; *n* = 50). (D) Amplitude of the sural nerve in relation to age at the time of examination (*r*_s_ = −0.461; *P* = 0.001; *n* = 50). **Significant group difference of sural sNAP in the groups Hoehn and Yahr 1–3 versus Hoehn and Yahr 4–5 calculated by Mann‐Whitney‐U test with *P* < 0.01.

**Table S1.** Normal values for electrophysiology by Stöhr et al.^17^

**Table S2.** Correlation analyses of nerve conduction studies and high‐resolution ultrasound.
