# Supplementary figures and images for: Correlates of polyneuropathy in Parkinson’s disease
Source: Ann Clin Transl Neurol. 2020 Sep 17;7(10):1898–907. doi: 10.1002/acn3.51182 (PMC7545593; doi:10.1002/acn3.51182)

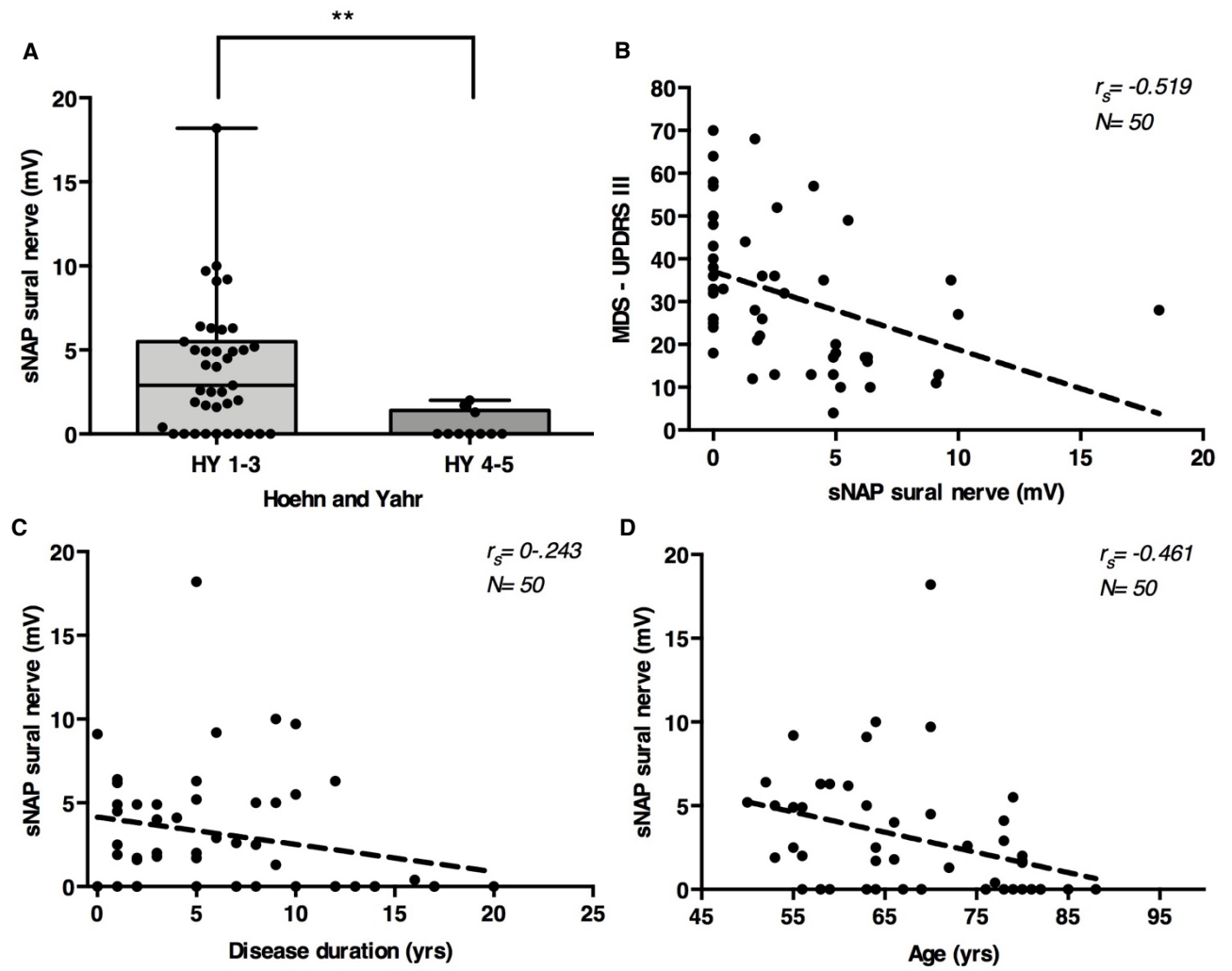

Supplementary Fig. 1

Supplement: Supplementary file 1 — Figure S1. (A) Amplitude of the sural nerve in Hoehn and Yahr Groups (n = 50). (B) MDS‐UPDRS III in relation to the amplitude of the sural nerve (r s = −0.519, P < 0.001; n = 50). (C) Amplitude of the sural nerve in relation to disease duration (r s = −0.243; P = 0.088; n = 50). (D) Amplitude of the sural nerve in relation to age at the time of examination (r s = −0.461; P = 0.001; n = 50). **Significant group difference of sural sNAP in the groups Hoehn and Yahr 1–3 versus Hoehn and Yahr 4–5 calculated by Mann‐Whitney‐U test with P < 0.01. [file ACN3-7-1898-s001.pdf]
