# Supplementary material for: Correlates of polyneuropathy in Parkinson’s disease
Source: Ann Clin Transl Neurol. 2020 Sep 17;7(10):1898–907. doi: 10.1002/acn3.51182 (PMC7545593; doi:10.1002/acn3.51182)
Supplement: Supplementary file 2 — Table S1. Normal values for electrophysiology by Stöhr et al. 17 [file ACN3-7-1898-s002.docx]

Supplementary Table 1

|  | Sensory nerve action potential of the sural nerve (mV) | Compound motor action potential of the tibial nerve (mV) | Compound motor action potential of the tibial nerve (mV) | Sensory nerve action potential of the median nerve (mV) |
| --- | --- | --- | --- | --- |
| Cut-off | <50 years: <3.6  ≥50 years: <5.0 | <5.0 | <5.0 | <5.0 |
