# Supplementary material for: Correlates of polyneuropathy in Parkinson’s disease
Source: Ann Clin Transl Neurol. 2020 Sep 17;7(10):1898–907. doi: 10.1002/acn3.51182 (PMC7545593; doi:10.1002/acn3.51182)
Supplement: Supplementary file 3 — Table S2. Correlation analyses of nerve conduction studies and high‐resolution ultrasound. [file ACN3-7-1898-s003.docx]

|  | NCS Mean amplitude (mV)  N. suralis | NCS Mean amplitude (mV)  N. tibialis | NCS Mean amplitude (mV)  N. medianus (motor) | NCS Mean amplitude (mV)  N. medianus (sensory) |
| --- | --- | --- | --- | --- |
| HRUS mean CSA (mm²) of N. medianus (Carpal tunnel) | r_s_ = -0.055  p = 0.718  n = 45 | r_s_ = -0.040  p = 0.792  n = 45 | r_s_ = 0.183  p = 0.265  n= 39 | r_s_ = -0.145  p = 0.384  n= 38 |
| HRUS mean CSA (mm²) of N. ulnaris (Loge de Guyon) | r_s_ = 0.114  p = 0.455  n = 45 | r_s_ = -0.052  p = 0.735  n = 45 | r_s_ = 0.105  p = 0.526  n = 39 | r_s_ = 0.174  p = 0.297  n = 38 |
| HRUS mean CSA (mm²) of N. ulnaris (Sulcus ulnaris) | r_s_ = 0.045  p = 0.768  n = 45 | r_s_ = -0.072  p = 0.638  n = 45 | r_s_ = 0.186  p = 0.256  n = 39 | r_s_ = 0.108  p = 0.518  n = 38 |
| HRUS mean CSA (mm²) of N. fibularis (Caput fibularis) | r_s_ = 0.075  p = 0.629  n = 44 | r_s_ = 0.026  p = 0.854  n = 44 | r_s_ = 0.312  p = 0.056  n = 38 | r_s_ = 0.093  p = 0.583  n = 37 |
| HRUS mean CSA (mm²) of N. medianus  (upper arm) | r_s_ = -0.057  p = 0.711  n = 45 | r_s_ = 0.109  p = 0.476  n = 45 | r_s_ = 0.317  p = 0.049  n = 39 | r_s_ = 0.038  p = 0.820  n = 38 |
| HRUS mean CSA (mm²) of N. ulnaris (upper arm) | r_s_ = 0.062  p = 0.685  n = 45 | r_s_ = -0.056  p = 0.714  n = 45 | r_s_ = 0.082  p = 0.618  n = 39 | r_s_ = -0.095  p = 0.572  n = 38 |
| HRUS mean CSA (mm²) of N. fibularis  (fossa tibialis) | r_s_ = 0.025  p = 0.872  n = 44 | r_s_ = -0.028  p = 0.856  n = 44 | r_s_ = 0.198  p = 0.234  n = 38 | r_s_ = 0.096  p = 0.572  n = 37 |

Supplementary Table 2: Correlation analyses of nerve conduction studies and high-resolution ultrasound.
